# Effect of COVID-19 on the Organs

**DOI:** 10.7759/cureus.9540

**Published:** 2020-08-03

**Authors:** Uday Jain

**Affiliations:** 1 Anesthesiology, San Mateo Medical Center, San Mateo, USA

**Keywords:** covid-19, cytokine storm, endothelial dysfunction, hypercoagulability, acute kidney injury, acute respiratory distress syndrome, interleukin, stroke, myocarditis, anosmia

## Abstract

The COVID-19 pandemic that first became apparent in Wuhan, China, is now infecting millions all over the world. This is a review of COVID-19's extensive effects on virtually all the organs. It causes inflammation, endotheliitis, vasoconstriction, hypercoagulability, and edema. Lymphocytopenia, elevated D-dimer, elevated fibrin degradation products (FDPs), and disseminated intravascular coagulation (DIC) are observed. Deep vein thrombosis (DVT), venous thromboembolism, pulmonary embolism (PE), systemic and pulmonary arterial thrombosis and embolism, ischemic stroke, and myocardial infarction (MI) are reported.

In the heart it can cause acute coronary syndrome, congestive heart failure, myocarditis, and arrhythmias. Kidney injury is usually secondary to systemic abnormalities. Stroke occurs even in young patients. Delirium and seizures are common. Anosmia and impaired sense of taste are reported. Psychological problems are common among patients as well as providers. Stool may contain virus. Lactate dehydrogenase may be elevated. Various skin manifestations including patchy erythematous rash are reported.

## Introduction and background

The COVID-19 pandemic first became apparent in Wuhan, China. It has rapidly spread to all continents. In persons who develop clinical illness in response to SARS-CoV-2, the respiratory system is the most commonly affected. However, the virus can affect any organ in the body. In critically ill patients, multiple organs are often affected. The virus binds to angiotensin converting enzyme 2 (ACE2) receptors present in vascular endothelial cells, lungs, heart, brain, kidneys, intestine, liver, pharynx, and other tissue [1]. It can directly injure these organs. In addition, systemic disorders caused by the virus lead to organ malfunction. While managing a patient it is essential to evaluate for injury to multiple organs. Disturbances of coagulation and vascular endothelium are common but may not lead to symptoms in early stages. They contribute to injury to multiple organs. Cardiac and renal dysfunction is common among the patients who die. Injury to the organs may become apparent long after the acute infection has subsided. Different organs may be affected at different times. Chronic injury may occur. Rehabilitation can be long and difficult.

## Review

Inflammation and endotheliitis

Compared to other health conditions, COVID-19 can lead to a much greater production of cytokines by white blood cells [2]. A surge of catecholamines may precede and contribute to cytokine storm, also called as hypercytokinemia or cytokine release syndrome. This maladaptive response can lead to systemic inflammatory response syndrome (SIRS), acute respiratory distress syndrome (ARDS), multi-organ injury, shock, and death. Inflammatory response may continue to increase even when the viral load is diminishing. 

SARS-CoV-2 infects the endothelial cells in multiple organs and causes diffuse lymphocytic endotheliitis, leading to vasoconstriction [3]. Accompanying inflammation, hypercoagulability, and edema cause hypoperfusion leading to organ ischemia. However, patients with pre-existing immune-mediated inflammatory disease being treated with anticytokine biologics and other immunomodulatory therapies are not at an increased risk due to COVID-19 [4].

Effect on coagulation

Bleeding is not common in COVID-19. Deep vein thrombosis (DVT), venous thromboembolism, pulmonary embolism (PE) and cor pulmonale, systemic and pulmonary arterial thrombosis and embolism, ischemic stroke and myocardial infarction (MI) are reported [5-10]. DVT and PE are common among the dead [7]. This is caused by inflammation, platelet activation, hypercoagulability, endothelial dysfunction, constriction of blood vessels, stasis, hypoxia, muscle immobilization, and disseminated intravascular coagulation (DIC) [5-10].

Fever and inflammation cause hypercoagulability and impair fibrinolysis. Cytokine interleukin-6 (IL-6) levels correlate with hypercoagulability and disease severity. Elevated antiphospholipid antibodies are associated with thrombosis [11]. The liver increases production of procoagulant substances. Prothrombin time and activated partial thromboplastin time are moderately prolonged. Moderate thrombocytopenia is observed. C-reactive protein is elevated. Cytokine storm and excessive systemic inflammation are associated with lymphocytopenia, elevated D-dimer, elevated fibrin degradation products (FDPs), and DIC. D-dimer levels and DIC are prognostic.

Guidelines recommend thromboprophylaxis [12-13]. Prophylaxis with low-molecular-weight or regular heparin, fondaparinux, or a direct oral anticoagulant such as apixaban or rivaroxaban should be considered. Heparins bind tightly to COVID-19 spike proteins impeding the entry of the virus into cells. Heparins also downregulate IL-6 and reduce immune activation. A nonrandomized study suggests that among patients requiring mechanical ventilation, systemic anticoagulation may be associated with reduced mortality without increasing major bleeding [14]. However, systemic anticoagulation has not proven to be beneficial in ARDS due to other etiologies. After hospital discharge extended prophylaxis may be beneficial.

Pulmonary effects

Autopsy studies indicate that in the acute phase the patients have classic diffuse alveolar damage without organization and fibrosis [15-16]. It is caused by disruption of endothelial and alveolar cells. This leads to fluid and cellular exudation and hyaline membrane formation. Acute fibrinous and organizing pneumonia are also observed [17]. It consists of alveolar fibrin aggregation. Airway inflammation is present. Increased capillary permeability causes alveolar and interstitial edema. Vascular angiogenesis is a distinguishing feature of COVID-19 [18-19].

On chest CT, findings of subpleural and peripheral areas of ground-glass opacity and consolidation are present in patients with COVID-19. Most of the patients have bilateral distribution. On chest radiographs, patchy infiltrates are observed that may be distributed asymmetrically.

Several modalities are available for managing respiratory insufficiency [20-23]. Oxygen via high-flow nasal cannula, and noninvasive ventilation are among the therapies utilized in these patients. Prone positioning may improve oxygenation [24-28].

Cardiac effects

In COVID-19, cardiac complications can precede and can occur in the absence of pulmonary and other complications [29-30]. Ischemic cardiac injury can occur in patients with established coronary artery disease (CAD), those with latent CAD, and those without CAD. The primary cause of the former two is plaque rupture and thrombosis. The last one is due to inadequate oxygen supply and mimics a MI. For acute coronary syndrome due to plaque rupture, antiplatelet and anticoagulation therapy may be beneficial. Fibrinolytic therapy and percutaneous coronary intervention may be considered. However, the reported incidence of acute MI has declined in the COVID-19 period [31].

Invasion of myocytes by the virus is observed in some patients. Systemic inflammatory response such as cytokine storm can cause myocarditis without direct viral infiltration. It can cause heart failure and arrhythmias. This can occur even after the acute phase of the infection has resolved and in the absence of lung damage.

About one-half of the non-survivors have acute cardiac injury and heart failure. Respiratory failure dominates in the early phases of the disease whereas cardiac injury becomes more critical in the later phases. Vascular risk factors of diabetes, obesity, age, and hypertension have greater association with mortality than does respiratory disease. In Britain one-quarter of the COVID-19 deaths occurred among diabetics, whereas 15% occurred among patients with chronic pulmonary disease.

Heart failure and elevation of brain-type natriuretic peptide (BNP) is observed. Elevated troponin and BNP levels are associated with mortality. PE can cause elevation of troponin as well as BNP. For older patients with existing CAD or hypertension, heart failure may be caused by worsening demand-supply relationship. Myocarditis is more likely the cause in younger patients. Arrhythmias include tachycardia, bradycardia, and asystole. They can be due to inflammation, myocarditis, hypoxemia, metabolic abnormalities, or medications.

Cardiovascular complications may occur long after viral clearance and recovery. Inflammation can persist and evolve silently. As an example, dyslipidemia, pulmonary fibrosis, and avascular necrosis evolved over the long term in many survivors of severe acute respiratory syndrome (SARS), which is closely related to COVID-19. 

Commonly used medications including angiotensin converting enzyme inhibitors and angiotensin II receptor blockers have not been demonstrated to increase the risk of COVID-19 infection or its complications and should not be discontinued [32].

Renal effects

COVID-19 complicates the management of patients on dialysis and with kidney transplantation [33]. In Britain about 15% of the patients who expired had chronic kidney disease. ACE2 receptors are present in kidneys [34]. The virus is found in glomerular cells, tubular epithelium, and podocytes of kidneys. Acute kidney injury (AKI) is commonly secondary to systemic abnormalities including diabetes, hypertension, chronic kidney disease, hypoxemia, and coagulopathy. Cytokine storms can cause drastic hypoperfusion and AKI. 

 Acute kidney injury is also caused by rhabdomyolysis due to hyperventilation or medications including antivirals such as remdesivir. In New York, about 90% of patients who were on mechanical ventilation developed AKI [35]. AKI occurs in temporal association with respiratory failure.

Due to shortage of continuous renal replacement therapy and other hemodialysis equipment and supplies, there is greater utilization of peritoneal dialysis. The latter is suboptimal in hospitalized patients, especially if they are unstable. The catheter for peritoneal dialysis is usually placed in the anterior abdomen. It is less effective in patients who are being proned because of respiratory failure. Placing the catheter on the side of the abdomen alleviates the problem.

Among kidney transplant recipients, initially fever is present in only about one-half and diarrhea is present in about one-quarter of the patients [36]. Compared to a matched cohort they have a faster progression of disease and a higher mortality.

Effect on brain

The ACE2 receptors are present in the cerebral cortex and brain stem. Some patients have meningitis and encephalitis indicating viral invasion of the central nervous system (CNS). There is depression of brain stem reflexes including the one that senses oxygen starvation. Neurological manifestations may be the only ones observed or may occur in combination with respiratory or other symptoms [37-39]. Neurological manifestations are more common in people with more severe disease. Altered oxygen and carbon dioxide levels may contribute to them. They include dizziness, headache, impaired consciousness including confusion, delirium, and inability to rouse. Delirium is common and can lead to long-term cognitive impairment including memory deficits. Because of a shortage of commonly used sedatives like propofol and dexmedetomidine, benzodiazepines are being used for sedation. They can enhance delirium. Brains of dead patients demonstrate hypoxic changes but encephalitis or other changes due to the virus are rare [40]. 

Cytokine storm can cause brain inflammation and edema. Some patients have sympathetic storm that can cause seizure-like symptoms. Stroke due to blockage of a cerebral artery can occur even in young patients with no prior history [41]. This is in part because of hypercoagulability and endothelial injury. Cerebral hemorrhage is also observed. Ataxia and seizure may be present. Cranial nerves may be involved. Anosmia and dysgeusia, i.e., impaired sense of taste, are reported [42]. Nerve pain, skeletal muscle weakness and pain, tingling or numbness in the hands and feet are also observed. Rhabdomyolysis may cause elevated serum creatine kinase. Neurological features among patients in ICU with ARDS included encephalopathy, agitation, and confusion [37]. Corticospinal tract signs with enhanced tendon reflexes, ankle clonus, and bilateral extensor plantar reflexes are present in most of the patients. 

Effect on eyes

Both ACE2 receptors and TMPRSS2 proteases that are necessary for infection by SARS-CoV-2 are found in ocular surface cells in cornea, inside the eyelids and in the white of the eye [43]. About one-third of hospitalized patients develop ocular abnormalities including conjunctivitis [44]. Conjunctivitis is more common in the sicker patients. Ocular involvement may occur early. Ocular surface cells are portals of entry and reservoirs of the virus. Ocular virus shedding is a source of infection. Infectious virus can persist in the eye for up to three weeks [45].

Gastrointestinal effect

Gastrointestinal (GI) symptoms include loss of appetite, nausea, vomiting, diarrhea, and abdominal pain or discomfort [46-47]. These symptoms might start before or occur with or without other symptoms such as fever, myalgias, and cough. Lower GI tract is rich in ACE2 receptors. Some patients’ stool contains intact infectious virus or only RNA and protein fragments of the virus. Patients who have virus in the stool take longer to clear it. Although a small percentage of patients have GI symptoms, up to one-half shed virus in the stool [47]. Virus protein shell is also found in gastric, duodenal, and rectal cells. More than one half of COVID-19 hospitalized patients have elevated lactate dehydrogenase and other liver enzymes indicating injury to the liver or bile ducts. This is likely to be due to an overactive immune system or due to drugs causing liver damage.

Effect on skin

Skin manifestations of COVID-19 are similar to those of other viruses and chronic inflammatory diseases like acne, eczema, psoriasis, and rosacea. Vascular problems associated with skin manifestations can be neurogenic, microthrombotic, or immune complex mediated. Of the patients with skin manifestations, a majority have patchy erythematous rash [48-49]. Some have widespread urticaria or hives. A few also have chickenpox-like fluid-filled vesicles or blisters. They can have measles-like rashes. The most commonly affected area is the trunk. Itching is mild or absent. Some patients have skin eruptions at symptom onset, and others after hospitalization. Lesions usually heal in a few days. Skin manifestations do not correlate with the severity of COVID-19.

Patients may develop livedo reticularis. It is a purplish net-like discoloration of the skin, often a result of blood clotting abnormalities. Lacy, dusky rashes, including dead skin cells are observed on the arms, legs, and buttocks. They are associated with hypercoagulability. Petechiae are present. Nonpruritic blanching livedoid vascular eruption, possibly due to vaso-occlusion may be present. They appear as mottled, net-like red, or pink patches. Also present are chilblains, which are purplish, slightly firm and often tender. COVID toes and fingers have frostbite-like areas with red or purple rash or hive-like eruption.

Psychological effects

Because of financial difficulties and social isolation due to COVID-19, many psychological problems can arise. They can be delayed by months. There is an increase in "deaths of despair" from substance abuse or suicide. The risk is greater among persons with dementia, mental illness, and autism. In person and online communication with friends and support professionals is beneficial.

On discharge from ICU, a third of the patients have dysexecutive syndrome consisting of inattention, disorientation, or poorly organized movements in response to command [37]. Some patients who recover from COVID-19 develop mental health problems [50]. These include anxiety, depression, and post-traumatic stress disorder (PTSD). Long term effects can include development of Alzheimer’s or Parkinson’s disease.

## Conclusions

SARS-CoV-2 virus binds to ACE2 receptors present throughout the body and can adversely affect virtually every system of the body. It can cause cytokine storm which can culminate in death. Different organs may be affected in different patients, in a temporal course unrelated to viral load. Inflammation, platelet activation, hypercoagulability, endothelial dysfunction, constriction of blood vessels, stasis, hypoxia, and muscle immobilization contribute to the complications.

The lungs are commonly affected. Acute coronary syndrome, heart failure, and myocarditis may be present. Patients who are on angiotensin converting enzyme inhibitors and angiotensin II receptor blockers should continue taking them. AKI is usually secondary to systemic derangements. Meningitis, encephalitis, encephalopathy, stroke, and delirium are observed. Impaired sense of smell and taste are observed. Eyes are sites of virus entry and can also be sources of infection. Psychological problems are common among patients as well as providers. GI symptoms are observed. Patchy erythematous rash is the most common skin manifestation. Thus, COVID-19 can affect virtually any organ in the body.

## References

[1] Wadman M, Couzin-Frankel J, Kaiser J, at al (2020). How does coronavirus kill? Clinicians trace a ferocious rampage through the body, from brain to toes. https://www.sciencemag.org/news/2020/04/how-does-coronavirus-kill-clinicians-trace-ferocious-rampage-through-body-brain-toes.

[2] Merad M, Martin J (2020). Pathological inflammation in patients with COVID- 19: a key role for monocytes and macrophages. Nat Rev Immunol.

[3] Varga Z, Flammer A, Steiger P (2020). Endothelial cell infection and endotheliitis in COVID-19. Lancet.

[4] Haberman R, Axelrad J, Chen A (2020). Covid-19 in immune-mediated inflammatory diseases — case series from New York. N Engl J Med.

[5] Bikdeli B, Madhavan M, Jimenez D (2020). COVID-19 and thrombotic or thromboembolic disease: Implications for prevention, antithrombotic therapy, and follow-up. J Am Coll Cardiol.

[6] Nahum J, Morichau-Beauchant T, Daviaud F (2020). Venous thrombosis among critically ill patients with coronavirus disease 2019 (COVID-19). JAMA.

[7] Wichmann D, Sperhake J, Lütgehetmann M (2020). Autopsy findings and venous thromboembolism in patients with COVID- 19: a prospective cohort study. Ann Intern Med.

[8] Lax S, Skok K, Zechner P (2020). Pulmonary arterial thrombosis in COVID-19 with fatal outcome: results from a prospective, single-center, clinicopathologic case series. Ann Intern Med.

[9] Ackermann M, Verleden S, Kuehnel M (2020). Pulmonary vascular endothelialitis, thrombosis, and angiogenesis in Covid-19. N Engl J Med.

[10] Creel-Bulos C, Hockstein M, Amin N (2020). Acute cor pulmonale in critically ill patients with Covid-19. N Engl J Med.

[11] Zhang Y, Xiao M, Zhang S (2020). Coagulopathy and antiphospholipid antibodies in patients with Covid-19. N Engl J Med.

[12] (2020). Coronavirus disease 2019 (COVID-19) treatment guidelines. https://covid19treatmentguidelines.nih.gov.

[13] Alhazzani W, Møller M, Arabi Y (2020). Surviving sepsis campaign: guidelines on the management of critically ill adults with coronavirus disease 2019 (COVID-19). Intensive Care Med.

[14] Paranjpe I, Fuster V, Lala A (2020). Association of treatment dose anticoagulation with in-hospital survival among hospitalized patients with COVID-19. J Am Coll Cardiol.

[15] Barton L, Duval E, Stroberg E (2020). COVID-19 autopsies, Oklahoma, USA. Am J Clin Pathol.

[16] Xu Z, Shi L, Wang Y (2020). Pathological findings of COVID-19 associated with acute respiratory distress syndrome. Lancet.

[17] Copin M, Parmentier E, Duburcq T (2020). Time to consider histologic pattern of lung injury to treat critically ill patients with COVID-19 infection. Intensive Care Med.

[18] Hariri L, Hardin C (2020). Covid-19, angiogenesis, and ARDS endotypes. N Engl J Med.

[19] Ackermann M, Verleden S, Kuehnel M (2020). Pulmonary vascular endothelialitis, thrombosis, and angiogenesis in Covid-19. N Engl J Med.

[20] Marini J, Gattinoni L (2020). Management of COVID-19 respiratory distress. JAMA.

[21] Patel B, Kress J, Hall J (2020). Alternatives to invasive ventilation in the COVID-19 pandemic. JAMA.

[22] Wang K, Zhao W, Li J (2020). The experience of high-fow nasal cannula in hospitalized patients with 2019 novel coronavirus-infected pneumonia in two hospitals of Chongqing, China. Ann Intensive Care.

[23] Ferreyro B, Angriman F, Munshi L (2020). Association of noninvasive oxygenation strategies with all-cause mortality in adults with acute hypoxemic respiratory failure. A systematic review and meta-analysis. JAMA.

[24] Sarma A, Calfee C (2020). Prone positioning in awake nonintubated patients with COVID- 19: necessity is the mother of invention. JAMA Intern Med.

[25] Thompson A, Ranard B, Wei Y (2020). Prone positioning in awake, nonintubated patients with COVID-19 hypoxemic respiratory failure. JAMA Intern Med.

[26] Telias I, Katira B, Brochard L (2020). Is the prone position helpful during spontaneous breathing in patients with COVID-19?. JAMA.

[27] Elharrar X, Trigui Y, Dols A (2020). Use of prone positioning in nonintubated patients with COVID-19 and hypoxemic acute respiratory failure. JAMA.

[28] Sartini C, Tresoldi M, Scarpellini P (2020). Respiratory parameters in patients with COVID-19 after using noninvasive ventilation in the prone position outside the intensive care unit. JAMA.

[29] Madjid M, Safavi-Naeini P, Solomon S (2020). Potential effects of coronaviruses on the cardiovascular system: a review. JAMA Cardiol.

[30] Akhmerov A, Marbán E (2020). COVID-19 and the heart. Circ Res.

[31] Solomon M, McNulty E, Rana J (2020). The Covid-19 pandemic and the incidence of acute myocardial infarction. N Engl J Med.

[32] Vaduganathan M, Vardeny O, Michel T (2020). Renin-angiotensin-aldosterone system. Inhibitors in patients with Covid-19. N Engl J Med.

[33] Alberici F, Delbarba E, Manenti C (2020). Management of patients on dialysis and with kidney transplantation during the SARS-CoV-2 (COVID-19) pandemic in Brescia, Italy. Kidney Int Rep.

[34] Puelles V, Lütgehetmann M, Lindenmeyer M (2020). Multiorgan and renal tropism of SARS-CoV-2. N Engl J Med.

[35] Hirsch J, Ng J, Ross D (2020). Acute kidney injury in patients hospitalized with COVID-19. Kidney Int.

[36] Akalin E, Azzi Y, Bartash R (2020). Covid-19 and kidney transplantation. N Engl J Med.

[37] Helms J, Kremer S, Merdji H (2020). Neurologic features in severe SARS-CoV-2 infection. N Engl J Med.

[38] Lahiri D, Ardila A (2020). COVID-19 pandemic: a neurological perspective. Cureus.

[39] Mao L, Jin H, Wang M (2020). Neurologic manifestations of hospitalized patients with coronavirus disease 2019 in Wuhan, China. JAMA Neurol.

[40] Solomon I, Normandin E, Bhattacharyya S (2020). Neuropathological features of Covid-19. N Engl J Med.

[41] Oxley T, Mocco J, Majidi S (2020). Large-vessel stroke as a presenting feature of Covid-19 in the young. N Engl J Med.

[42] Spinato G, Fabbris C, Polesel J (2020). Alterations in smell or taste in mildly symptomatic outpatients with SARS-CoV-2 infection. JAMA.

[43] Zhou L, Xu Z, Castiglione G (2020). ACE2 and TMPRSS2 are expressed on the human ocular surface, suggesting susceptibility to SARS-CoV-2 infection. BioRxiv.

[44] Wu P, Duan F, Luo C (2020). Characteristics of ocular findings of patients with coronavirus disease 2019 (COVID-19) in Hubei province, China. JAMA Ophthalmol.

[45] Colavita F, Lapa D, Carletti F (2020). SARS-CoV-2 isolation from ocular secretions of a patient with COVID-19 in Italy with prolonged viral RNA detection. Ann Intern Med.

[46] Han C, Duan C, Zhang S (2020). Digestive symptoms in COVID-19 patients with mild disease severity: clinical presentation, stool viral RNA testing, and outcomes. Am J Gastroenterol.

[47] Parasa S, Desai M, Chandrasekar V (2020). Prevalence of gastrointestinal symptoms and fecal viral shedding in patients with coronavirus disease 2019. A systematic review and meta-analysis. JAMA.

[48] Darlenski R, Tsankov N (2020). Covid-19 pandemic and the skin - what should dermatologists know?. Clin Dermatol.

[49] Recalcati S (2020). Cutaneous manifestations in COVID‐ 19: a first perspective. J Eur Acad Dermatol.

[50] Pfefferbaum B, North C (2020). Mental health and the Covid-19 pandemic. N Engl J Med.

